# Molecular and Physiological Properties Associated with Zebra Complex Disease in Potatoes and Its Relation with *Candidatus* Liberibacter Contents in Psyllid Vectors

**DOI:** 10.1371/journal.pone.0037345

**Published:** 2012-05-17

**Authors:** Veria Y. Alvarado, Denis Odokonyero, Olivia Duncan, T. Erik Mirkov, Herman B. Scholthof

**Affiliations:** 1 Department of Plant Pathology and Microbiology, Texas A&M University, College Station, Texas, United States of America; 2 Texas AgriLife Research, Texas A&M System, Weslaco, Texas, United States of America; United States Department of Agriculture, United States of America

## Abstract

Zebra complex (ZC) disease on potatoes is associated with *Candidatus* Liberibacter *solanacearum* (CLs), an α-proteobacterium that resides in the plant phloem and is transmitted by the potato psyllid *Bactericera cockerelli* (Šulc). The name ZC originates from the brown striping in fried chips of infected tubers, but the whole plants also exhibit a variety of morphological features and symptoms for which the physiological or molecular basis are not understood. We determined that compared to healthy plants, stems of ZC-plants accumulate starch and more than three-fold total protein, including gene expression regulatory factors (*e.g*. cyclophilin) and tuber storage proteins (*e.g*., patatins), indicating that ZC-affected stems are reprogrammed to exhibit tuber-like physiological properties. Furthermore, the total phenolic content in ZC potato stems was elevated two-fold, and amounts of polyphenol oxidase enzyme were also high, both serving to explain the ZC-hallmark rapid brown discoloration of air-exposed damaged tissue. Newly developed quantitative and/or conventional PCR demonstrated that the percentage of psyllids in laboratory colonies containing detectable levels of CLs and its titer could fluctuate over time with effects on colony prolificacy, but presumed reproduction-associated primary endosymbiont levels remained stable. Potato plants exposed in the laboratory to psyllid populations with relatively low-CLs content survived while exposure of plants to high-CLs psyllids rapidly culminated in a lethal collapse. In conclusion, we identified plant physiological biomarkers associated with the presence of ZC and/or CLs in the vegetative potato plant tissue and determined that the titer of CLs in the psyllid population directly affects the rate of disease development in plants.

## Introduction

The presence of potato psyllids and their associated disorders were first documented in the 1920's in Colorado, and described as psyllid yellows disease of solanaceous plants, displaying upward cupping of leaves and dwarfing of the plants [1]. The disease was considered to be caused by a toxin or a virus, but at that time no conclusive causal agent was reported. Since then anecdotal evidence suggested that certain disease symptoms might be associated with the presence of phytoplasma in some instances [2]. However, evidence is increasing that the potato psyllid *Bactericera cockerelli* transmits an α-proteobacterium *Candidatus* Liberibacter solanacaerum (CLs) to several solanaceous crops causing two disorders named after their effects in the plants; psyllid yellows (PY) and zebra chip or zebra complex (ZC) disease [3]. ZC has recently become an important economical disease in Texas [4], currently spreading northward in the USA [5] causing increased monetary losses to the potato industry. A similar triangular association between psyllids, a CL species, and plants, is thought to cause citrus greening [6]. In addition to being a serious disease on potatoes, because of the much faster host generation time and wider geographic distribution, once sufficiently understood, experimental ZC systems could potentially serve as a research model for studying molecular plant-microbe-psyllid interactions associated with citrus greening.

ZC disorder is named after its characteristic stripe discoloration pattern observed in the potato chips after frying [5], decreasing their marketable value. The present consensus is that the occurrence of ZC is closely associated with the presence of CLs in plants and in transmitting psyllids [7], [8]. In this context correct identification presents technical challenges because CLs is a phloem restricted α-proteobacterium which at present is non-culturable [9], [10] Currently, detection of CLs in the potato plant and psyllids is only feasible by the amplification of a conserved 16S ribosomal DNA fragment through either conventional PCR [11], [12], nested PCR [13] or qPCR [14]. The bacteria accumulate to relatively high levels in the plant roots [14], perhaps as a result of the natural flow of nutrients (source to sink) in the phloem. However, the uneven distribution of CLs through the entire plant oftentimes prevents detection of the bacteria by PCR in foliar tissue [10] even when symptoms reminiscent of ZC are observed. Therefore, in the present study we implemented PCR-based methods in combination with the chip frying test to permit the selection of only those plants positive for both tests for subsequent analysis.

Characteristic above ground symptoms of ZC vary from mild to severe but these can easily be confused with symptoms caused by other pathogens [11]. Some commonly observed ZC-associated symptoms include: curling up of emerging leaves, purple and yellow discoloration of the new shoots, leave scorching, stem thickening, occasional formation of aerial tubers, zigzagging of the stem, tuber and chip discoloration and early senescence of the plant [5]. In spite of all these symptoms, only the chip discoloration that is readily identified upon frying is the most reliable and routinely used method by scientists and the potato industry to determine if the disorder observed in the field is due to ZC. Therefore, our first objective was to define molecular biomarkers to better understand the underlying molecular physiological principles of ZC disease.

The potato psyllid vector of ZC (and CLs) is a phloem feeding insect that requires the presence of obligate endosymbionts to acquire essential amino acids not present in the plant sap [15]. Along with these obligate or primary endosymbionts, psyllids carry facultative or secondary endosymbionts, which can establish differential symbiotic relationships. For instance, we noticed variations in psyllid prolificacy for three different psyllid populations of the same biotype maintained in the laboratory [16], which was corroborated by findings that levels of CLs in psyllids affected population growth rate and longevity [17]. These findings suggest that CLs act as secondary endosymbiont to confer different fitness characteristics to the psyllids [17]. A recent study on the diversity of endosymbionts in the potato psyllids found that the primary endosymbionts *Candidatus* Carsonella ruddii and *Wolbachia* were present in all developmental stages of the insect [18]. Considering these properties, our second objective was to develop RT-qPCR and traditional PCR-based methods to precisely monitor CLs population dynamics and primary endosymbiont levels in individuals of different ZC-transmitting psyllid colonies, and to determine if a direct relationship exists between any of these and colony prolificacy or the severity of disease in plants.

The collective goal of the aforementioned two objectives was to obtain a better understanding of the potato-psyllid-CLs molecular interactions responsible for the onset of ZC disease. In order to begin to dissect the triangular complex we analyzed samples from the field and those grown under laboratory conditions with and without ZC using PCR to identify truly CLs-positive samples. These were then used to determine the nature of molecular host responses and physiological changes caused by infection. Also, we newly established and maintained several psyllid colonies under controlled experimental conditions for transmission studies with a focus on two psyllid populations that contain either relatively low or high titers of CLs. Using these identified ZC affected plants and CLs-transmitting psyllid colonies, the studies revealed the new findings that: i) stems of potato plants infected with CLs acquire tuber-like properties such as accumulation of starch and tuber-enriched proteins, ii) these stems have increased levels of total phenolic compounds as well as elevated quantities of polyphenol oxidase enzyme, iii) the level of CLs in psyllids and its effect on prolificacy is not related to differences in endosymbiont titers, and iv) the CLs levels in the psyllid insect vector directly correlate with the degree and rate of symptom induction in the ZC affected plants.

## Results

### Plant physiological properties associated with ZC disease

#### Sample selection

**Figure 1:**
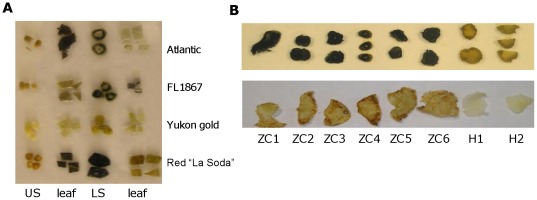
Detection of starch accumulation by lugol staining. **A**. Typical starch accumulation pattern in different plant tissues of healthy potato varieties. Upper portion of the stem (US), lower portion of the stem (LS). **B**. Lugol staining of the upper stem (US) of potato FL1867 chipping variety affected by ZC. H1 and H2 are the healthy controls. The results of frying test performed on tuber slices performed are shown in the lower panel.

An initial screening of field-collected samples was conducted to verify presence of CLs and potato chip brown discoloration pattern. Conventional PCR was used to identify a CLs specific 16S ribosomal DNA sequence [11], and a frying test (Fig. 1) was conducted to corroborate if the potatoes had the defect that characterizes ZC. Only samples positive for both tests were then subjected to further analysis.

#### Abnormal starch accumulation

Barriers in the plant phloem can cause sugar levels to increase in the stems of potato plants and induce the formation of starch storage organelles known as amyloplasts. For example, a macroscopically observable characteristic that could possibly result from such sugar accumulation in ZC plants is the formation of aerial tubers, something also observed during potato infections with fungus *Rhizoctonia solani* on the lower part of the stem [19]. Because ZC disease is associated with the presence of CLs, and this phloem-restricted α-proteobacterium can potentially form a barrier to normal photosynthate flow, we hypothesized that in ZC plants photosynthate nutrient distribution is impaired resulting in the accumulation of starch in the stem tissues. To address this, we first tested different potato varieties to determine the normal pattern of starch accumulation by the standard lugol staining test, and identified that healthy potato plants accumulate starch at varying degrees in different plant organs, except for the upper stems (Fig. 1). Then we proceeded and compared accumulation of starch in the upper stems of healthy and ZC affected plants and correlated that with the appearance of ZC-typical dark discoloration in fried tuber chips. As hypothesized, starch was accumulating abnormally in the upper stems of ZC-affected plants while this did not occur in healthy controls. This characteristic was proven to consistently appear in all ZC diseased plants.

#### Protein content

At the onset of the studies we noticed that plants diseased with ZC develop a fleshy and succulent stem instead of the characteristic hollow and brittle stem of healthy potato plants of the Atlantic and FL1867 chipping varieties. This characteristic was observed in all different types of potato samples whether grown in the field or under laboratory conditions. To evaluate whether the observable changes in stem structure related to differences in total protein content, infected tissues from plants collected in the field were subjected to standard Bradford assays. Healthy control plants were from the same immediate area where the ZC affected plants were collected. The protein quantification was performed with 0.5 g of fresh tissue and values are presented as mg/g of tissue in Table 1. The difference in protein content was significant based on a one way ANOVA and a Tukey's test, P value of 0.01. On average stems of the diseased potato plants contained 3.5 times more protein per gram of tissue when compared to healthy potato plants.

**Table 1:** Protein content of healthy and ZC affected plants.

Sample	Protein* (mg/g)	SD**
Healthy-1	1.04	0.072
Healthy-2	1.24	0.072
Healthy-3	0.95	0.082
Healthy-4	1.21	0.273
Healthy-5	0.46	0.090
Healthy-6	1.39	0.170
Healthy-7	1.43	0.020
ZC-1	2.88	0.112
ZC-2	3.06	0.055
ZC-3	3.37	0.097
ZC-4	6.42	0.116
ZC-5	4.38	0.121
ZC-6	4.03	0.047

* Bradford assays were used to determine the protein concentration of healthy and ZC affected stem tissue. The plant samples were measured in three replicates.

** A statistical analysis one-way ANOVA using SPSS 14.0 and Tukey mean comparison with P = 0.01 showed that the ZC diseased plants have a significant increase of protein content per gram of tissue compared to healthy potato plants. The potato plants were sampled in the field and were of different varieties (FL1867, Norkotah or Norgold).

#### Protein profile

**Figure 2:**
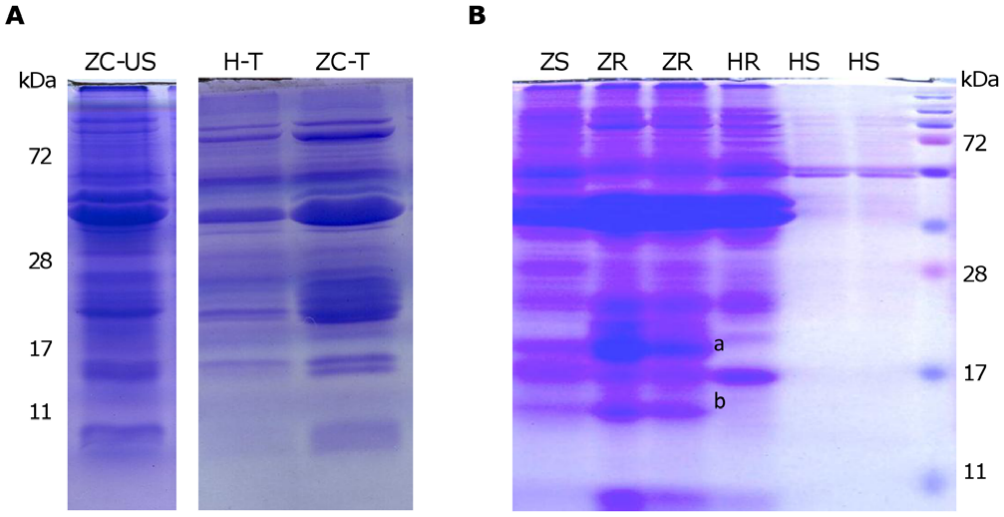
SDS-PAGE of proteins stained with Coomasie brilliant blue. **A**. Profile comparison of upper stem (US) of ZC diseased plants with tubers (T) of healthy or ZC plants. **B**. Profile comparison of the root (R) and stem (S) tissue of ZC diseased (Z) and healthy (H) plants, a and b depict the differentially expressed proteins; (a) contains two proteins, cyclophilin and a putative transcription factor BTF-3 and (b) represents glycoprotein-like product. Approximately 7.5 ug of protein per lane were loaded and run in a 15% polyacryalmide gel, size markers in kDa are shown on the edges.

Protein profiling is a broad molecular screening technique that can be used to identify major proteins that accumulate abundantly in diseased tissue in plants either as a result of plant (defense) responses or produced by the invasive microbe. For this study, total proteins were extracted from several healthy and ZC diseased potato plants and their protein profiles compared using standard SDS-PAGE. The results showed that ZC affected stem tissues displayed a protein profile resembling a healthy potato tuber (Fig. 2A). Furthermore, we identified differentially expressed proteins by Coomassie brilliant blue and silver staining of samples run on 15% polyacrylamide gels. Two specific protein bands present in infected plants were excised and sent for MALDI-TOF mass spec identification (Fig. 2B). Three proteins were specifically detected only in ZC affected plants, protein band-a contained: i) a basal transcription factor BTF-3 like, and ii) a single domain cyclophilin protein, and protein band-b represented, iii) a glycoprotein-like protein. These proteins were not of pathogen origin or defense response related proteins, but rather they appeared to represent host proteins involved in general processes of transcription and translation; events that seem to be actively up-regulated in the symptomatic ZC potato stems.

#### Cyclophilin

**Figure 3:**
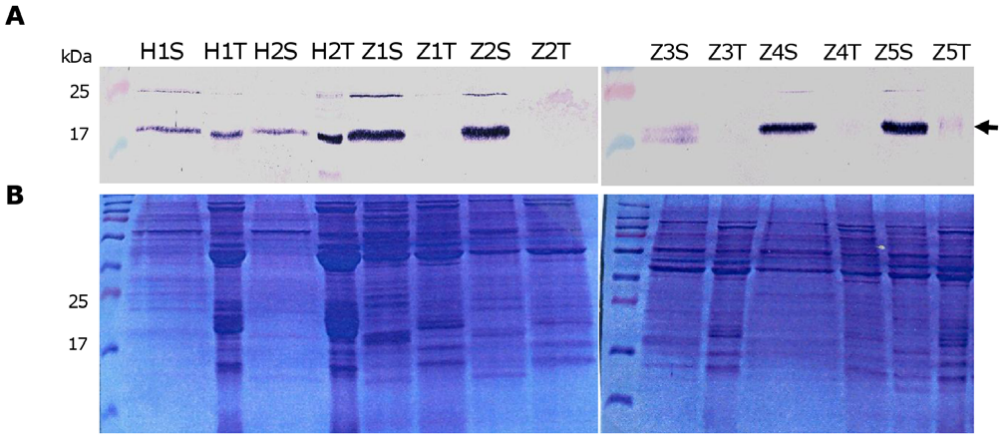
Cyclophylin detection. A *Solanum sogarandinum* cyclophilin antibody was used for detection of cyclophylin in ZC and healthy potato plants. **A.** Western blot analysis showing cyclophilin (17.5 kDa, indicated by arrow) detection in both stem (S) and tubers (T) of healthy (H) tissues, but no detection in tubers of Zebra complex (Z) diseased plants. **B.** Coomassie brilliant blue stained gel of the protein samples used for the western blot in A, the protein concentration was calculated by Bradford assay and equal amounts of proteins (7.5 ug) were loaded. Size markers are indicated.

The presence of the peptidyl-prolyl isomerase single domain cyclophilin protein was verified with immuno (western) blotting experiments. For this purpose a cyclophilin specific antibody was used [20]. This specific antibody successfully recognized the cyclophilin protein in the potato samples (Fig. 3A). Comparative western blot analysis with several healthy and ZC affected field samples corresponding to the stem or the tubers revealed that cyclophylin was present at elevated levels in the stems of plants afflicted by ZC when compared to healthy control plants (Fig. 3). Moreover, cyclophilin was only absent from the ZC infected tubers (Fig. 3B) which seem to be in a developmental stage that resembles older tissues, where no active protein production is occurring. Aging studies on this aspect were performed on potato tubers, corroborating the notion that this particular cyclophilin plays roles in actively translating tissues since only the potatoes that were not aged expressed cyclophilin (data not shown).

#### Patatins

**Figure 4:**
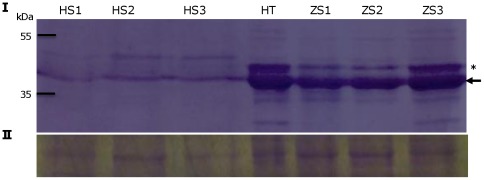
Detection of patatin proteins in healthy and ZC potato plants. **I.** Alkaline phosphatase western blot using a patatin-specfic antibody crossreacting with two bands; a class I patatin of 40 kDa (arrow) and a class II patatin (*), size markers in kDa. **II.** Coomasie loading control. HS represents healthy plant stem; HT, healthy tuber and ZS, ZC plant stem.

Mass spec protein identification of proteins in the previous section also revealed the presence of a number of patatin like protein fragments in healthy and ZC plants. Class I patatins are the major storage proteins that can be detected in potato tubers and are readily observed on a Coomassie brilliant blue stained gel, migrating at around 40 kDa, [21], also class II patatins can be detected in potato tubers although they compose up to 30% of the total pool of patatin storage proteins [22]. Therefore, we tested the hypothesis of organ identity change of the ZC stem by assaying presence or absence of patatin proteins via western blot analysis on potato samples collected from the field. The results demonstrated that class I and II tuber-specific patatin antibody reactive proteins were abundantly present in healthy tuber and stems of ZC affected plants, once more indicating a change in the molecular programming of ZC afflicted stems (Fig. 4).

#### Phenolic compounds and polyphenol oxidase

**Figure 5:**
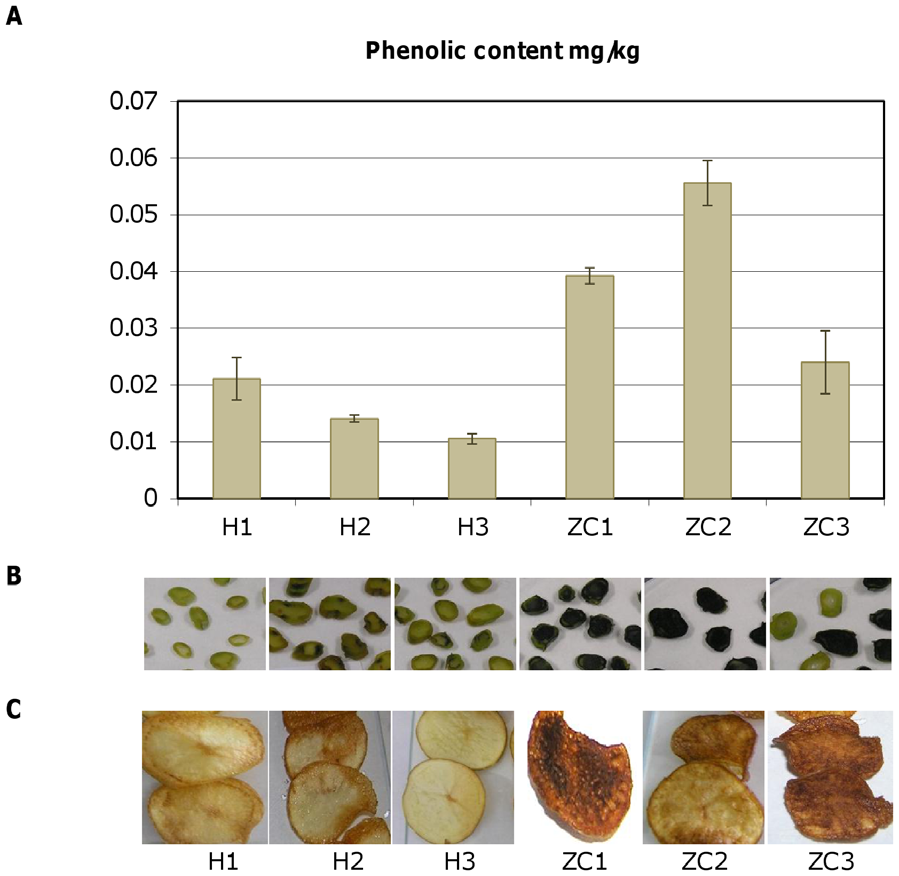
Analysis of total phenolic compounds in ZC potato stems. **A**. Total phenolic compounds were analyzed using the Folin-Ciocalteu method. **B**. Results of lugol staining on the stems of the tissues used for phenolic content assays. **C**. Results of frying test performed on tuber slices of the same plants samples used in B. H, healthy stem and chips, ZC, ZC diseased.

Compared to healthy plants, tissue from ZC-affected potato plants rapidly displays a brown discoloration upon protein extraction. This can be attributed to an abundance of phenolic compounds in the stem of ZC affected plants or to an increase in polyphenol oxidase activity because when using reducing agents or antioxidants in the extraction buffer, such as β-mercaptoethanol or DTT, the brown discoloration is not observed, while browning readily occurs upon extraction with TE buffer. To address this, we analyzed the samples from healthy and ZC diseased plants using a standard total phenol extraction method (Fig. 5). A selection of the most representative samples was made based on PCR, lugol staining of stems and frying test of tuber slices (Fig. 5B–C), and these samples were then evaluated for total phenol content. A two-fold increase of total phenols was found in ZC stems when compared to healthy stems (Fig. 5A). Polyphenol oxidase (PPO) is the enzyme involved in the phenol oxidation and its content was assayed via western blot analysis. The results pointed to a clear increase of oligomeric PPO amounts in ZC-diseased plants (Fig. S1). These findings indicate that ZC-affected tissues are characterized by increasing levels of phenol substrate and PPO to result in the formation of polyphenolic compounds that cause typical ZC-associated brown discoloration.

### Characterization of microbiota in psyllids

#### Establishment of psyllid colonies

The varying degrees of symptoms and syndromes (*e.g*. psyllid yellows) caused by psyllids on potato plants, lead us to initiate psyllid transmission studies in order to understand plant responses to psyllid feeding compared to infestation with psyllids containing different levels of CLs. Different psyllid colonies of the same biotype initially either containing no detectable CLs, or high levels of the bacteria, were established and maintained in the laboratory in cages with potato and tomato plants that were periodically replaced by new plants. The insect colonies were frequently evaluated for CLs concentration using PCR techniques, as described in subsequent sections.

#### Monitoring of CLs in individual psyllids

**Figure 6:**
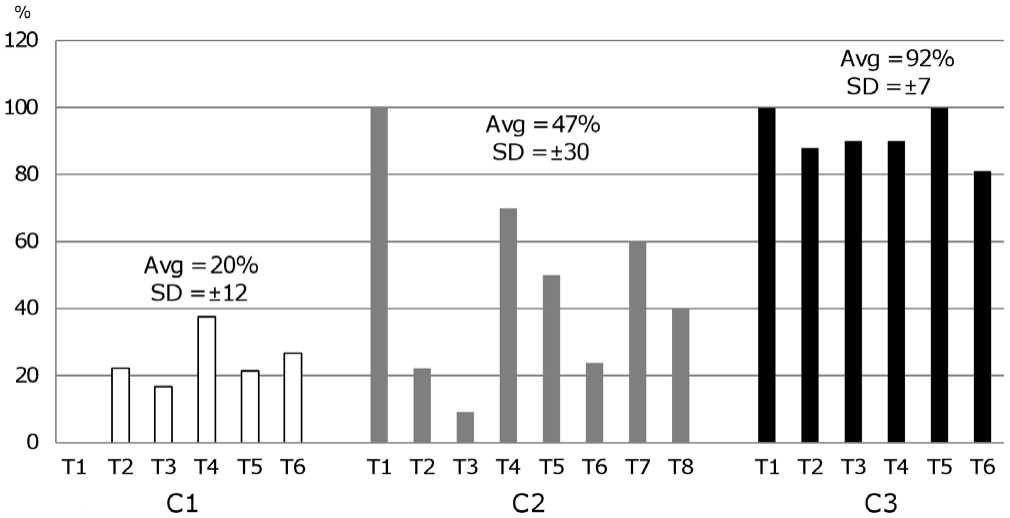
Temporal characterization of psyllid colonies. Percentage of psyllids positive for the presence of *Candidatus* Liberibacter *solanacearum* (CLs) were determined by conventional PCR of a 16S rDNA specific region at irregular time points (T1 to T8). The colonies evaluated were C1, a psyllid colony with low titer of CLs, and C2 and C3, which were known to contain higher titers of CLs. The average (Avg) and the standard deviation (STDEV) for each colony were calculated and are shown above each group of time points. Y axis, percentage of positive psyllids; X axis, time points. Sample dates separated by one or more days.

Conventional PCR was first used to screen different psyllid colonies for the presence or absence of CLs. DNA extractions of single psyllids were performed with C-TAB and the PCR screening was done with CLs specific primers, ZCf/OI2c, [11], and with 28S psyllid ribosomal DNA primers, designed to be used in multiplex PCR for a DNA quality test [11]. We identified an insect colony (C1) with generally undetectable levels of CLs in individual psyllids, but sometimes testing positive (at relative low titers) in a low percentage of adults and nymphs (Fig. 6). During the course of the study a more sensitive nested primer set [13], was used to routinely monitor the relatively low CLs titer C1 psyllid colony. A second psyllid colony (C2) tested positive for CLs in an average of ∼47% of the individuals tested (adults and nymphs), whereas the third colony (C3) tested positive for 92% of its population (Fig. 6). Another important observation was that in a high percentage of CLs-positive psyllids generally correlated with high titers in individual psyllids (Fig. S2) which had a negative impact on psyllid prolificacy (Fig. S3). In fact this effect of continuous elevated CLs levels in a high percentage of C3 individuals on prolificacy led to the eventual demise of the colony.

One more finding that surfaced as a result of the periodic PCR screening for CLs was that in contrast to the relatively stable high percentage and titer of CLs in psyllids of the C3 colony (92%, SDEV ±7), the percentage of psyllids containing detectable levels of CLs in the laboratory colony C2 temporally fluctuated (between 9% to 100%). Therefore, extra sampling was required in order to have a better representation of the proportion of C2 psyllids that carried CLs. This variability of CLs is illustrated by the high SD (±30) obtained for this psyllid colony (Fig. 6). Based on these analyses it was clear that frequent monitoring was pertinent and that we did not possess a colony absolutely free of CLs because during the course of this study even the C1 colony contained at least some individuals within the population that tested positive for CLs. Therefore, for all subsequent experiments we categorized newly established colonies as low-CLs or high-CLs based on the combination of the percentage of psyllids testing positive and the relative level in individuals (Fig. S2).

#### Microbe population dynamics in psyllids

Based on recurrent observations it was noticed that the low-CLs C1 psyllid population showed a high rate of prolificacy and outnumbered the high-CLs C2 psyllid population (Fig. S3). In order to begin to understand the microbial population dynamics and the changes in reproduction rates of these two psyllid colonies, we investigated potential changes in population densities of other microbes such as *Wolbachia spp*. The underlying premise was that this secondary endosymbiont is known to alter reproduction behaviors in different insects [23] and is present in the potato psyllid [18], [24], and therefore it was considered possible that CLs-mediated changes in its endosymbiont density could potentially affect psyllid reproduction. To assess such possible changes we developed a semi-quantitative SYBR green based real-time PCR method that allowed us to assess CLs levels in individual psyllids and compare those to levels of other endosymbionts such as *Candidatus* Carsonella *ruddii*, a primary psyllid endosymbiont [18], [25], and two *Wolbachia* strains. Primers for the 28S psyllid ribosomal DNA reference gene, the endosymbionts *Carsonella* and the two strains of *Wolbachia* present in potato psyllids, were successfully designed (Table S1) and complied with the real time qPCR standard MIQE guidelines for primer efficiency to allow reliable comparisons [26].

**Figure 7:**
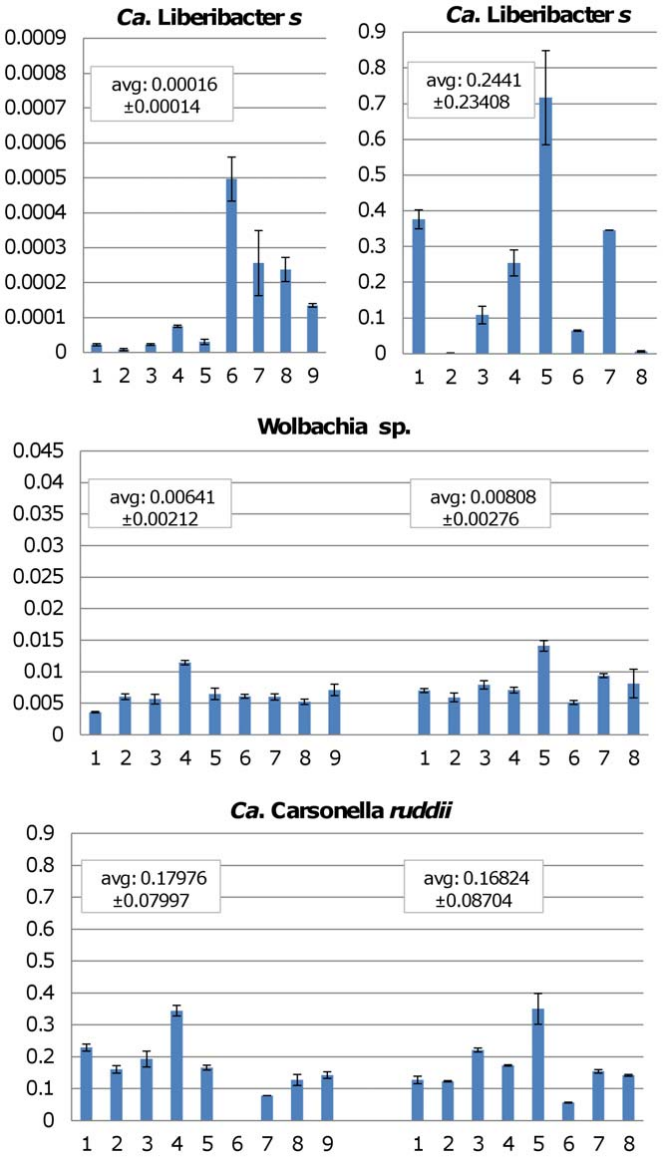
Relative expression of specific gene amplicons by RT-qPCR. Columns represent the different microbial titers in the potato psyllid vector and bars denote average values of three technical repeats for individual psyllids normalized to psyllid 28S rDNA. C1 and C2 correspond to the psyllid colonies with low- and high-CLs abundance, respectively. Notice that the values for the *Ca*. Liberibacter *solanacearum* in colony 1 (C1) are extremely low and therefore are shown on a 1/1000 fold scale compare to the other graphs. The average and SDEV for each group of samples were calculated and shown in boxes above their respective graphs. A statistical analysis one-way ANOVA using SPSS 14.0 and Tukey mean comparison with P = 0.01 showed that there are significant statistical differences in the CLs titers of C1 and C2 psyllid colonies, but no differences between titers of *Candidatus* Carsonella *ruddi* or *Wolbachia sp*.

Based on the qPCR analysis on the two psyllid populations (C1 and C2) no differences were apparent for *Candidatus* Carsonella ruddii or in the population of *Wolbachia sp*. Both psyllid colonies contained the same relative amounts of these microbes (based on Tukey's test P value of 0.01, Fig. 7), while the relative amount of CLs were evidently superior and statistically different (Tukey's test P value of 0.01) in the high-CLs C2 psyllid colony (Fig. 7). As in previous experiments, for unknown reasons, considerable CLs titer variation was observed among individuals evaluated in both colonies (Fig. 7, note the vastly different scales on y-axis for CLs level in C1 and C2 on the top panel). Together these analyses demonstrated that the microbial dynamics in the low-CLs or high-CLs psyllid populations do not obviously affect the populations of the two major endosymbionts present in psyllids, *Carsonella* and *Wolbachia*.

### Plant-CLs-psyllid interactions

#### Plant responses upon colonization of potatoes with psyllids

The need to understand the spectrum of ZC symptoms observed in potato plants lead us to begin psyllid transmission studies with insect populations carrying low or high titers of CLs, and examine the plant responses to varying exposure times. Towards this, the first transmission studies initially aimed at dissecting the contribution of psyllid infestation were performed in the greenhouse with a C1 psyllid colony that at the beginning did not contain detectable levels of CLs. Atlantic potato plants, known to be quite susceptible [8] were planted in caged pots and 6 weeks later 40 psyllids were released into each cage. The psyllids were exterminated after 5 and 12 weeks (treatment I and II respectively) of insect exposure. For both treatments, psyllids and plant tissues were collected right before insecticide application and conventional PCR and nested PCR were used to determine the presence of CLs in individual psyllids and in plants. Consistent with results presented in the previous section, the C1 colony was not free of CLs, and in fact the results showed that 53% of psyllids were CLs-positive for treatment I at 5-w, and for treatment II 26% at 5-w and then further increased to 38% at 12-w, as shown in Table 2. However, as noted before, despite these percentages of positive C1 psyllids the titer of α-proteobacteria in each individual was still relatively low (example in Supplemental Fig. S2), for instance only the use of nested primers allowed the detection of CLs at 12-w for treatment II (Table 2).

**Table 2:** Percentage of C1 psyllids with detectable levels of CLs.

Treatments*	Conventional PCR % of psyllids	Nested PCR % of psyllid
I-5 weeks	6.0	53.0
II-5 weeks	13.0	26.0
II-12 weeks	0.0	38.0

* Transmission studies were performed with the low-CLs psyllid colony. A conventional and nested PCR was used to assess CLs percentage in sampled population at 5 and 12 weeks of plant exposure to psyllids.

**Figure 8:**
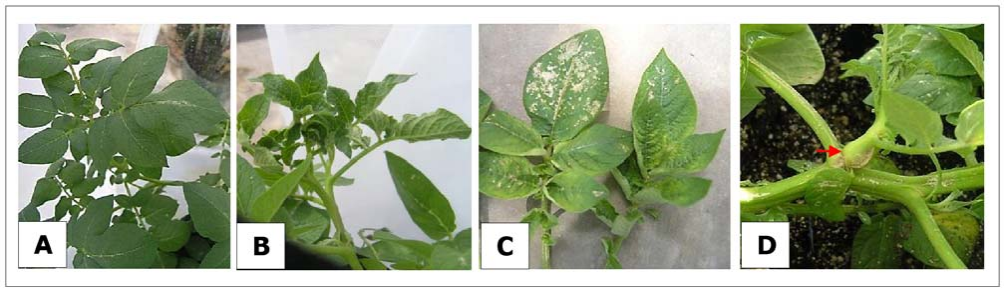
Symptoms of potato plants exposed to C1 psyllids with a low–CLs content. Differences between caged plants with or without psyllids containing low amounts of CLs. **A** shows a caged control potato plant, non-exposed to psyllids and **B, C** and **D** are potato plants exposed to low-CLs psyllids. Symptoms observed vary from curling up of new leaves (B), Psyllid yellows-like symptoms (C) and formation of aerial tubers (red arrow, D).

**Figure 9:**
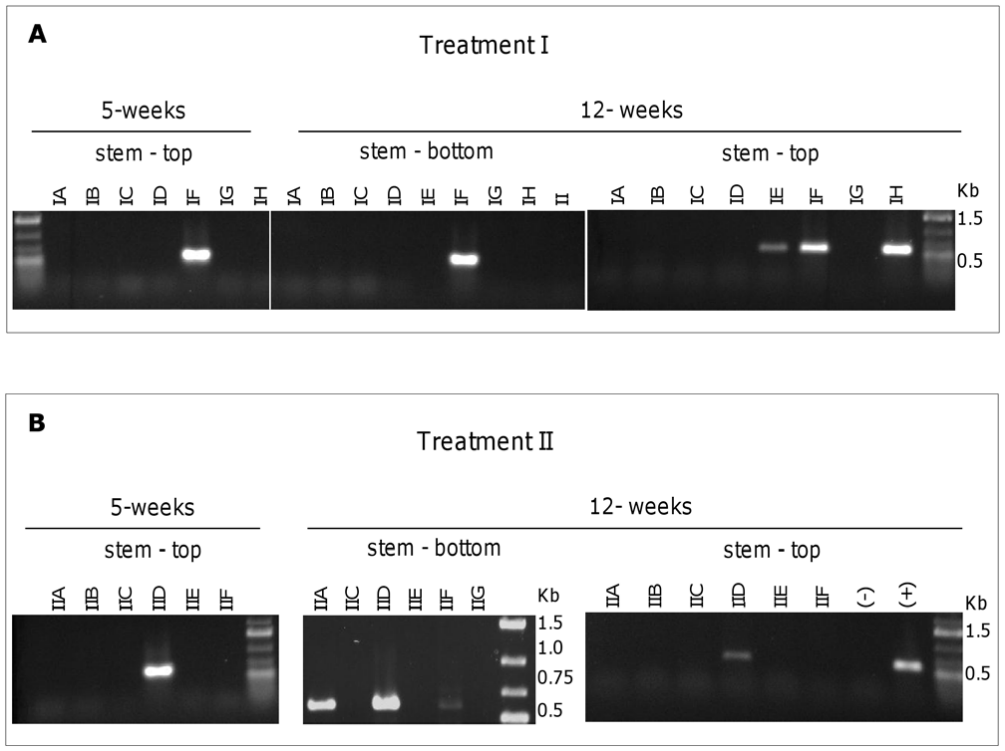
Nested PCR screening of plants exposed to low-CLs psyllids. Nested PCR for the amplification of a 580 bp 16S rDNA amplicon was conducted in all DNA samples for Treatment I and II. Plant tissue was collected at 5 and 12 weeks from the initiation of the experiment. IA – IH and IIA-IIF, DNA samples of plants exposed to psyllids; samples II and IIG, DNA samples of control plants non-exposed to psyllids. Molecular markers are indicated in kilo bases (Kb).

For both time-points the plants developed PY-like symptoms, such as curling up of new leaves, yellowing of the leaf blade and the appearance of some aerial tubers (Fig. 8). Lugol staining was performed on the upper portions of the stems and all plants that were infested with psyllids tested positive for starch accumulation (Table 3). However, none of the stem pieces used for DNA extraction were positive for CLs with the conventional multiplex PCR method (Fig. S4A) and even though only one plant sample showed cyclophilin accumulation (Fig. S4), patatin was abundantly expressed in the plants exposed with these C1 psyllids (data not shown). A nested PCR was also run on these stem DNA samples, confirming that two of the plant samples contained CLs for treatment I (Fig. 9A) and three plant samples were CLs-positive for treatment II (Fig. 9B), in other words, C1 psyllids were transmitting CLs to the plants but due to the low titers and uneven distribution not all plants were positive. Tubers were not fully developed at the time of harvest, due to heat stress conditions that were not optimal for tuber development. Interestingly, upon potato chip slicing we observed symptoms of heat necrosis only in potato plants that were not exposed to psyllids, and upon frying no typical ZC patterns were observed for any treatment. Collectively, these experiments suggested that exposure of potato plants to C1 psyllids initially harboring no CLs, but containing low levels of CLs towards the end, did acquire low quantities of CLs that caused some symptoms consistent with ZC but not the full range, suggesting a correlation between levels of CLs and host responses culminating in disease symptoms.

**Table 3:** Lugol staining of potato stems.

Treatment	Collection time	Sample	Lugol staining
I	5 wks.	A–H	+
		Control (I–J)	−
	12 wks.	A–H	+
		Control (I–J)	−
II	5 wks.	A–F	+
		Control (H)	−
	12 wks.	A–F	+
		Control (H)	−

Potato stem - tops were sliced and reacted with lugol solution to determine presence of starch. Positive (+) indicates that tissue reacted with the lugol solution displaying a dark blue color, and negative (−) indicates that the tissue remained the same color.

#### Effect of CLs abundance in psyllid colonies on plant survival

To more directly examine the effect of CLs inoculum titer on disease symptom severity, a second set of experiments was conducted under controlled growth conditions with low-CLs (C1) and high-CLs (C2) psyllid colonies, including a long term exposure to low-CLs psyllids. Six-week old Atlantic potato plants were placed in cages and inoculated with 20 low-CLs C1 or high-CLs C2 psyllids per plant, two plants per cage and placed in lighted shelves in the laboratory with 24°C constant temperature and 12 hours of light. A caged control contained plants without psyllids. In the short-time exposure experiments, two and a half weeks later the psyllids were exterminated. The long exposure experiments were conducted with the C1 colony on Atlantic potato plants (20 days old) exposed to psyllids for nine weeks, in growth chambers with 18 hours light/25°C and 6 hours dark/22°C.

Insects were sampled to assess the levels of CLs during the transmission studies by conventional PCR (Table 4). The intriguing finding even though consistent with previous experiments, was that the low-CLs C1 colony over time produced an increased number of psyllids with detectable levels of CLs, and surprisingly during the short time exposure experiment a higher number of psyllids (83%) tested positive for CLs compared to the psyllids that remained nine weeks on potato plants (27%). In comparison, the fraction CLs-positive psyllids in the high-CLs C2 colony was originally 100%, which decreased moderately to 69%, after 2.5 weeks (Table 4), while the CLs titers in individual positive psyllids remained quite high. Therefore, one set of plants could be considered having undergone relatively low exposure (C1) whereas the other was exposed from the onset to high levels of CLs (C2) by a combination of high percentage and titer of CLs and accompanying lower prolificacy than C1.

**Table 4:** Assessment of CLs in individual psyllids.

Treatments	Conventional PCR*% of psyllids
C1-0 weeks	21.0
C1–2.5 weeks	83.0
C1–9.0 weeks	27.0
C2-0 weeks	100.0
C2–2.5 weeks	69.0

* Percentage of psyllids positive for CLs are represented in this table.

**Figure 10:**
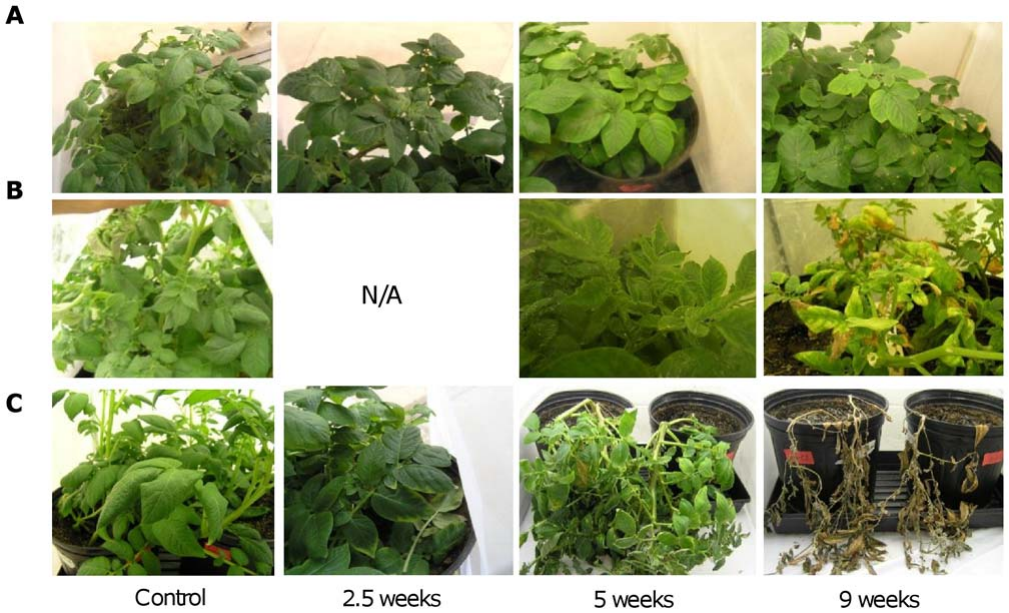
Disease progression on potato plants after psyllid exposure. **A.** Atlantic plants exposed for 2.5 weeks to low-CLs psyllids, no drastic symptoms were observed, and plants appear normal at the end of experiment. **B.** Atlantic plants exposed for 9 weeks with the low-CLs psyllid colony, disease progression is visible by leaf edges curling up proceeded by a strong psyllid yellows like symptoms. **C.** Atlantic plants exposed 2.5 weeks to high-CLs psyllids. At the time of insecticide treatment the plants have a normal appearance, but 2 weeks later the symptoms develop, the plant collapses and dies 3 weeks later. Plants caged without psyllids are labeled as control, and the time points shown (2.5, 5 and 9 weeks) are in weeks after psyllid inoculation.

As with the experiments in the greenhouse in the previous section, all plants infested with CLs-positive psyllids developed some degree of ZC symptoms (Fig. 10). Also consistent was that despite the presence of some level of CLs in the low-CLs psyllids, none of the plant samples exposed to these low-CLs insects tested positive for the presence of CLs when using multiplex or qPCR, and with one exception when using conventional PCR (Fig. S5). In contrast, even though the number of psyllids in the C2 and C1 colony were the same, the titers of CLs were different, therefore, plants infested with the C2 psyllids rapidly collapsed in a matter of weeks, consistent with the elevated CLs levels (Fig. S5). Consequently no potato tubers were recovered from these plants and screening of the plant tissue at the end of the experiment was also impossible since the plant material quickly decomposed (Fig. 10 C).

These findings provide supportive evidence for the conclusion that ZC transmission by low-CLs psyllids results in a very low overall CLs content and distribution in plants and consequently comparatively moderate symptoms are induced. However, transmission by high-CLs psyllids leads to rapid lethal disease symptoms probably due to multiple blockages along the phloem by the proteobacteria and explosive induction of host responses.

## Discussion

### Plant responses to CLs

Our studies showed that the intensity of physiological responses in potato plants to *Candidatus* Liberibacter solanacearum (CLs) associated with ZC, correlates with the content level of bacteria in the transmitting psyllid population. A variety of syndromes can be explained by the presence of different levels of CLs in the plants and the resulting number of sites that can possibly be physically blocked in the phloem by bacteria, their exudates or biofilms, to cause interruption of the flow of photosynthates. However, our experiments also demonstrate that part of the syndrome can be attributed to molecular reprogramming events that are caused by CLs. For instance, as illustrated by the lugol staining test, an abnormal accumulation of starch occurs in the upper stems of potato plants exposed to psyllids that transmit CLs.

The expression of the tuber storage protein patatin, can also be affected by changes in the photosynthate flow. It has been reported that patatins can be induced in petioles and stems when tubers and axillary buds are removed from potato plants, and also when potato leaves are incubated in high sucrose concentrations [27]. Furthermore, patatin expression seems to be regulated by STOREKEEPER, a DNA binding protein that is activated upon sucrose accumulation [28], [29]. Here, we observed an elevated accumulation of patatins and possibly other proteins, that are normally present in tubers but now accrue in the stems.

The overall protein content of ZC afflicted plants is also surprisingly high for such disease stressed plants. Up to 3.5 times more protein accumulates in ZC stems when compared to healthy stems, and again this could serve towards explaining the changes in tissue morphology and its contents. A protein profile comparison of ZC affected plants with healthy potato plants revealed that even though no proteins of microbial origin were identified, three host proteins were found to be enhanced in ZC affected plants. These were a peptidyl-prolyl isomerase single domain cyclophilin protein [20], a putative transcription factor BTF-3 like [30], [31] and a glycoprotein-like protein, which based on amino acid sequence similarity to Arabidopsis could be a 60S ribosomal protein L14, with roles in protein translation [32]. The increase of cyclophilin was verified with western blot analyses. The three proteins share a common function in transcription and translation; processes that seem to be actively turned on in the ZC diseased potato stems, which contain high amounts of total protein. The exact role of these putative regulatory protein remains to be identified but cyclophilins are known to stabilize the cis-trans transition state and accelerate isomerization, a process that is considered important in protein folding [33] and since overall protein levels are high, this would necessitate elevated levels of cyclophilin.

The rapid brown discoloration of sliced or ground tissue of ZC plants is likely caused by polyphenol oxidase (PPO), a copper metallo-enzyme that catalizes the oxidation of phenolic compounds to quinones, which upon polymerization react with amino acids on cellular proteins generating brown pigmentation in wounded tissues [34], [35]. PPO is localized in plastids, i.e. chloroplast and thylakoid lumen [36] and in potatoes it can be found in the amyloplast [37] where the starch granules concentrate. Upon tissue disruption, by mechanical damage or insect feeding the phenolic substrates that are accumulated in the vacuole will be brought in contact with the PPO released from the plastids, allowing the oxidation reaction to take place. Previous studies with tubers affected by ZC did show an increase in total phenol compounds but no assays were performed with the potato stems [38]. Here we show that stem tissues of ZC affected plants contain more phenolics than the healthy plants as well as higher levels of PPO Increasing phenolic compounds is an antimicrobial strategy that some plants use to restrict the growth or spread of the microbes [39], suggests that this accumulation of phenolic compounds and PPO in infected plants could be components of a plant defense response to the invasion of CLs bacteria.

### Plant-microbe-insect interactions

We have shown that several potato psyllid colonies have varying levels of CLs content and for reasons still unknown (*e.g*., environmental cues, food supply triggers, or sensing of psyllid population densities) the titers of the α-proteobacteria and percentage of CLs-positive individuals in colonies fluctuate over time and this affects the prolificacy of the insect colony. Moreover, in extreme cases we occasionally noted that the percentage of individuals testing positive would switch from a few to the majority and *vice versa* (Fig. 6). This phenomenon has also been observed for psyllid colonies maintained under greenhouse conditions (Don Henne, personal communication). Likewise, collaborators working with colonies derived from the same stocks as those used in our study, showed that levels of CLs in psyllids affect population growth rate and longevity [17]. Extrapolating these observations to what occurs in the field suggest the distinct possibility that in one year an exponential growth of low-CLs psyllids might occur but with low incidence of ZC, as was indeed observed in 2010 [40]. In other years there may be low numbers of high-CLs psyllids but a high incidence of ZC infected plants in the potato fields.

Microbial population dynamics were studied in relation to the fluctuation of CLs and differences in proliferation traits identified for the psyllids populations with different CLs contents. *Wolbachia ssp*, was the first suspect because of its known role in insect reproduction [23], [41], but the levels of these bacteria remained the same when comparing the evaluated low-CLs and high-CLs psyllid populations. Levels of *C*. Carsonella *ruddi* a primary endosymbiont [25] also remained similar for the two populations. Therefore, other factors or other microbes must influence the fitness properties, especially since the changes in CLs content can change within a matter of days (Fig. 6). In this context we are intrigued by the discovery that *Candidatus* Liberibacter *asiaticus* (CLa) associated with citrus greening, seems to harbor two phages which become lytic when CLa is injected into periwinkle plants [42]. It is known that during such lytic stages the phage destroys its bacterial host and one can imagine that similar changes to lytic stages could be responsible for the CLs titer fluctuation in psyllids. Sequences that resemble prophage genomes are present in the published CLs genome [43], and future experiments may elucidate if active phages are present within CLs.

Psyllid-mediated CLs transmission studies were conducted in order to better understand the underlying basis for the induction of different ZC responses. Our studies illustrated the existence of a clear correlation between CLs content levels in the psyllid population and severity of plant symptoms. For example, exposure of potato plants to psyllids containing high titers of CLs for two and a half weeks was sufficient under our conditions to cause plant death. Prolonged exposure time was a contributing factor to exacerbation of symptoms when psyllids with low CLs titer were used. Under such low infection pressure detection of CLs in the plants tissues is not always possible due to the low bacterial levels in combination with uneven distribution [9], [10], which affects the number of CLs-positive plants that can be detected (Fig. 9 and Fig. S4), but still we clearly observed the consequences of the bacteria infiltrating the phloem tissue as evidenced by disease symptoms. At the physiological and molecular level this was evidenced by the observations that for the most part plants exposed to CLs-harboring psyllids eventually accumulated starch in the upper stems, exhibited increased amount of protein per gram of tissue, contained elevated levels of the patatin storage protein, and phenolic content levels were higher. The severity or rate of symptoms onset culminating in the ZC syndrome is likely dependent on how fast the blockage of phloem occurs and to what extent the plants reprogram the stem identity and stem composition into a tuber-like condition.

### Summary

Several different physiological and molecular ZC-associated host responses were identified that include abnormal starch and protein accumulation in stems and induction of specific proteins, phenolics and PPO. We also established and maintained CLs-transmitting psyllid colonies, and developed SYBR green based real time qPCR procedures to determine the titer of CLs and other microbes in the potato psyllids. With these newly established properties, materials and tools; it was shown that the content levels of CLs in the psyllid populations can fluctuate over time for reasons unknown, but as demonstrated it is unlikely associated with variations on reproduction-associated endosymbionts. Importantly, the ultimate level of CLs at the time of inoculation appears to be key in the extent of disease symptom development in potato plants that at least partly results from physiological reprogramming events. High inoculum levels presumably allow for the rapid spread of the α-proteobaceria through the phloem causing a rapid wilt and collapse of potato plants. Under lower inoculum pressure plants disease incidence is lower, although if the CLs-transmitting psyllid colony persists on plants for several weeks, the effective size of the inoculum increases with time, resulting in severe symptoms and loss of the potato tuber production. Our results support the conclusion that physiological reprogramming events contribute to the ZC disease syndrome in potatoes and that the severity and rate of symptom development in plants correlate with the CLs inoculum density in the transmitting psyllids.

## Materials and Methods

### Plant material

Potato plants of the varieties Atlantic and FL1867 were grown in 2G pots, under a fertilizer regime of 8∶45∶14 NPK. Cages used for the transmission experiments were BugDorm-2120 insect rearing tents of 60×60×60 cm (MegaView Science Co., Ltd, Taichung, Taiwan). Plant materials (Atlantic, FL1867, Norkotah, Red La Soda) were also collected from the potato fields or obtained through Dr. Creighton Miller, for purposes of screening and characterization of plant physiological responses.

### Psyllid colonies

Three psyllid colonies were obtained from different sources. The C1 colony was obtained from Dalhart TX, generously provided by Drs. Charlie Rush and Don Henne. The C2 colony was obtained from Dr. Joe Munyanenza, Wapato Washington, and the C3 colony was generously provided by Dr. Christian Nansen, Lubbock TX. The psyllids are susceptible to Agrimek and Marathon insecticides, and these were used as the manufacturer recommended, whenever the psyllids needed to be exterminated (*i.e.* psyllid transmission studies). Psyllid colonies were maintained by periodic transfer of young tomato and potato plants in the Bug Dorm cage.

### Starch detection

Samples were placed in a tube containing I_2_/KI solution: (5 g KI, 0.5 g I_2_, 500 ml H_2_O), followed by an 80% ethanol wash. A positive reaction produced a dark blue precipitate in the tissue where the starch was accumulating.

### Phenotype analysis of chips

Potato tubers of different plants were rinsed, peeled and sliced. Slices of approximately 1.3 mm were cut by hand with a knife. The slices were rinsed in water and blot dried with paper towels. A deep fryer containing peanut oil (high flash point) was used to make the chips. When the oil temperature reached 355°F the chips were fried for 45 seconds. The chips were removed and placed on paper towels to eliminate the excess oil. Chips were then photographed.

### Protein extraction, quantification and immunoblot assays

For total protein quantification ∼0.5 g of tissue was extracted in 100 mM Tris-HCl pH8, 500 mM NaCl, 50 mM EDTA and 10 mM β-mercaptoethanol. Protein samples were then quantified using the Bradford method and a standard curve was obtained using BSA as a standard. Once the concentration of protein was calculated (mg/g of tissue) the numbers were then analyzed statistically using the program SPSS 14.0. For immunoblot assays, protein samples were electrophoresed through 10%, 12.5% or 15% polyacrylamide gels using SDS-PAGE and then electrotransferred to PVDF membranes. The blots were incubated with polyclonal rabbit antibodies raised against patatin [29] at 1∶2000, a gift from Dr. D.J. Hannapel (Iowa State University, IA), polyphenol oxidase [44] at 1∶800, kindly provided by Dr. L. Marqués (Université Montpellier 2, France), and cyclophilin [20] at 1∶3000, obtained from Dr. T. Rorat (Institute of Plant Genetics, Poland). Goat anti-rabbit IgG antibodies conjugated to alkaline phosphatase were used at 1∶3000 dilution, and the protein bands were then visualized by the addition of 5-bromo-4-chloro-3 indolyl phosphate *p*-toluidine and nitrotetrazolium blue salt.

### Total phenolic content

We followed an adapted Folin-Ciocalteu method [45]. Briefly, samples of 0.5 g were taken from stem tissue, ground in liquid nitrogen and mixed with 2 mL of 100% methanol, followed by a 24 h incubation at 4C. Equal amounts of sample and folic acid (62.5 uL) were mixed with 1 mL of dd water, vortexed and incubated for 2 min. Then 125 uL of sodium carbonate were added, mixed and incubated at room temperature for 2 hours. A standard curve was obtained using gallic acid as reference, and samples were measured at A_720_ in a Beckman-Coulter Spectophotometer Model DU 530.

### Detection of *Wolbachia* in psyllids by PCR

Psyllids were tested for the presence of *Wolbachia* by using the primer sets *wsp*-81F and *wsp*-691R [24]. The PCR reaction contained: 1× Phusion HF buffer, 200 µM dNTPs, 0.4 µM forward and reverse primer, 2 µl of DNA and 0.02 U of Phusion Hot Start DNA Polymerase. DNA amplification was performed on an Applied Biosystems 2720 Thermocycler following the conditions of 94°C (5 min), then 35 cycles of 94°C (30 s), 55°C (1 min), 72°C (1 min), and 72°C (5 min).

### PCR analysis

Conventional PCR and multiplex PCR were conducted as described before [11]. Modifications to Pitman's nested PCR protocol [13] were incorporated for our screenings. The primers used for the first round of PCR were ZCf/OI2c, and for the second round Lib 16S01F and Lib16S01R. Cycling parameters were also modified, the annealing temperatures for the first and second round were 62°C and 55°C respectively.

### Real-time qPCR

Primer design was based on specific sequences for *Bactericera cockerelli*, *Candidatus* Liberibacter *solanacearum*, *Carsonella ruddii* and *Wolbachia* obtained from the NCBI database. After PCR confirmation and amplicon sequencing, qPCR primers were designed with Primer Express 3.0 (Life Technologies Corporation, Carlsbad, CA). A PCR efficiency test was done to select the best primer pairs (Table S1). Briefly, curves for PCR amplification were established by a serial dilution of known psyllid DNA sample concentrations, and the efficiency was determined from the slope of the log linear portion of the calibration curve. The PCR efficiency equals (10^−1/slope^ -1). The theoretical maximum of 1 indicates that the amount of product doubles with each cycle. The real time PCR reaction was performed with 1× power SYBR green mix (Life Technologies Corporation), 500 nM forward and reverse primers and 30 ng of DNA sample in a 15 µl reaction. The PCR cycle used was as recommended (95°C for 10 minutes, 95°C for 15 seconds follow by 60°C for 1 minute for a total of 40 cycles). Ct values were obtained and then normalized to the 28S psyllid reference gene. The values relative to the reference gene were represented in graphs and the standard deviation of the technical repeats was determined.

## Supporting Information

Figure S1 **Detection of polyphenol oxidase (PPO).** Western blot analysis of polyphenol oxidase was performed with an apple PPO antibody (panel I) that successfully cross-reacted with the potato PPO. The Coomasie brilliant blue loading control of the protein samples used in the western blot is shown in panel II. The size of PPO is about 60 kDa [35]. The PPO enzyme is active as a tetramer, and as reported previously, some aggregated complexes can still be detected in an SDS-PAGE western blot [46] as seen in this western blot only for the ZC samples, indicated by arrow on right. HS, healthy stem; ZS, ZC affected stem; and ZT, ZC affected tuber.
(TIF)

Figure S2 **Analysis of CLs titer changes in psyllid populations.** The prolificacy of the psyllid colony is a very good indication of the density of CLs in the psyllids. The high-CLs psyllid colony (H-1 through H-3) with low prolificacy was used to initiate a new high-CLs colony, on a fresh set of tomato plants. However in the process of adapting to the fresh tissue in a new cage, the colony started to rapidly proliferate and when evaluated by PCR the density of CLs was very low. Subsequently a simultaneous screening on both psyllid populations was performed. Three psyllids per colony were randomly picked and DNA was extracted, including a “water” DNA extraction within sets to account for any possible contamination during the extraction. Conventional PCR was conducted with primer pairs Zcf/OI2c and 28SrDNA, used in a single PCR or combined in a multiplex. Results show that the prolific colony has reduced CLs titer (L-1 to L-3) and the initial high-CLs colony (H-1 to H-3) retained elevated amounts of CLs. Moreover when multiplex PCR was performed, only the high-CLs colony yielded comparable results, amplifying both PCR products, but the low-CLs sample did not produce a PCR product for the Zcf/OI2c primer pair, implicating that the primer ratios and conditions for multiplex PCR when testing low titer colonies need to be adjusted. Size markers in Kb.
(TIF)

Figure S3 **Prolificacy differences between psyllid colonies.** New colonies were established on potato plants with 5 pairs of female and male psyllids, after a month substantial differences in the population numbers were observed. **A.** High-CLs C3 colony, **B.** Low-CLs C1 colony.
(TIF)

Figure S4 **Molecular characterization of plants exposed to low-CLs psyllids.** **A**. Multiplex PCR was conducted on DNA extracted from stems of plants exposed to low-CLs psyllids. Arrows indicate the position of the 1,171 bp CLs and the 881 bp β-tubulin (β) amplicons. β-tubulin is used as a marker for DNA quality control, **B**. Total protein was extracted from the same tissues and western blot analysis was performed for the detection of cyclophilin (arrow). Sample loading is shown by the red Ponceau S staining. Molecular markers are indicated.
(TIF)

Figure S5 **Conventional PCR screening of plant samples exposed to low and high-CLs psyllids.** Conventional PCR tests were performed on DNA extracted from stems of plants exposed for 2.5 weeks to low-CLs (C1) and high-CLs (C2) psyllids. The arrow indicates the position of the 1.17 Kb ZCf/OI2c 16S rDNA amplicon. For the group of plants exposed to low-CLs, DNA samples A and B are from control plants that were caged without psyllids, and C through H represent plants exposed to psyllids, For plants exposed to high-CLs, A′ and B′ are DNA from control plants (caged without psyllids) and H′ and G′ are from plants exposed to high-CLs C2 psyllids. DNA ladder is indicated in kilo bases (Kb). Unnecessary lanes were removed (two lanes between marker and ZC plant DNA used as positive control).
(TIF)

Table S1 RT-qPCR primers.
(DOCX)
