# Supplementary figures and images for: Molecular and Physiological Properties Associated with Zebra Complex Disease in Potatoes and Its Relation with Candidatus Liberibacter Contents in Psyllid Vectors
Source: PLoS One. 2012 May 17;7(5):e37345. doi: 10.1371/journal.pone.0037345 (PMC3355140; doi:10.1371/journal.pone.0037345)

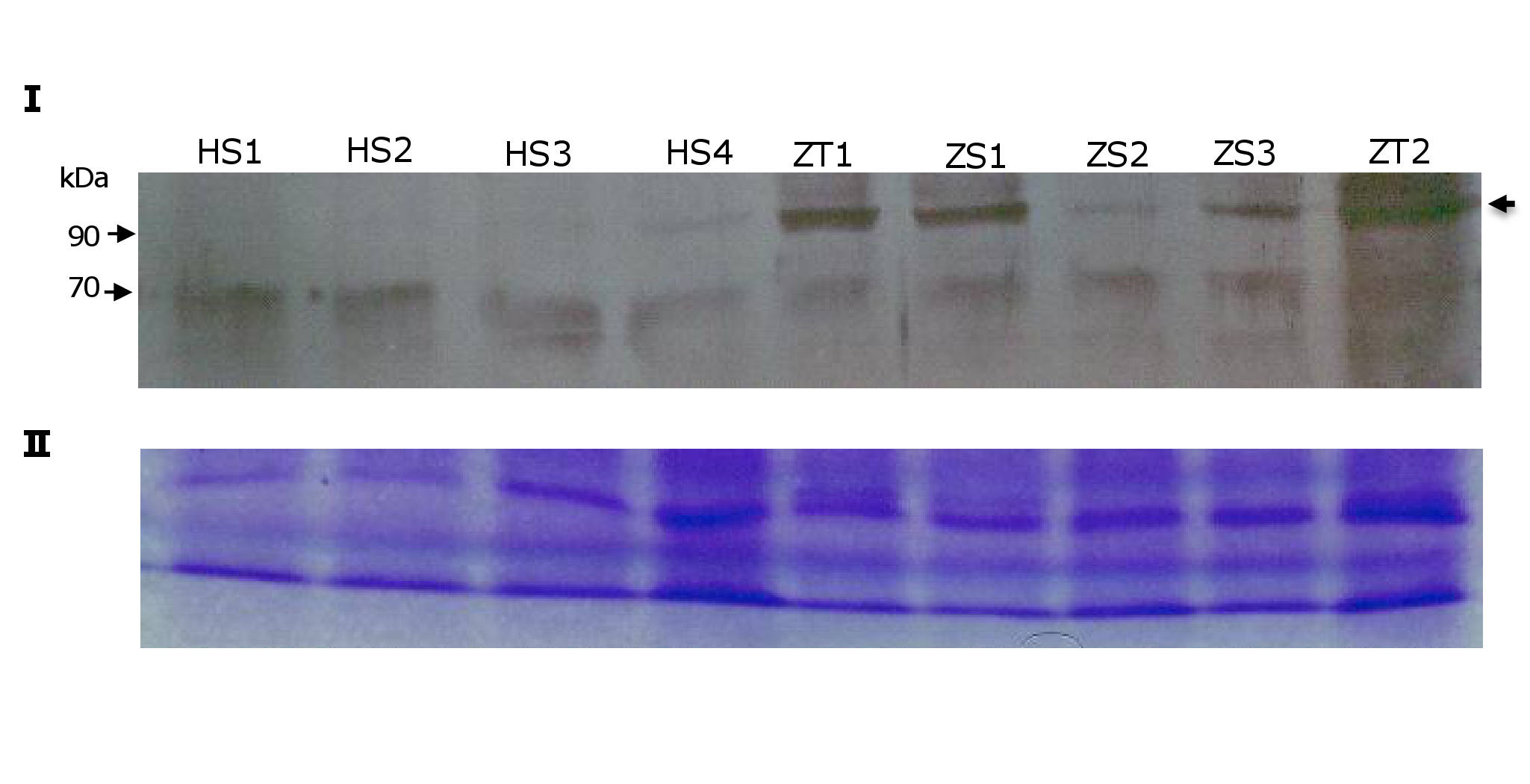

Supplement: Figure S1 — Detection of polyphenol oxidase (PPO). Western blot analysis of polyphenol oxidase was performed with an apple PPO antibody (panel I) that successfully cross-reacted with the potato PPO. The Coomasie brilliant blue loading control of the protein samples used in the western blot is shown in panel II. The size of PPO is about 60 kDa [35]. The PPO enzyme is active as a tetramer, and as reported previously, some aggregated complexes can still be detected in an SDS-PAGE western blot [46] as seen in this western blot only for the ZC samples, indicated by arrow on right. HS, healthy stem; ZS, ZC affected stem; and ZT, ZC affected tuber. (TIF) [file pone.0037345.s001.tif]

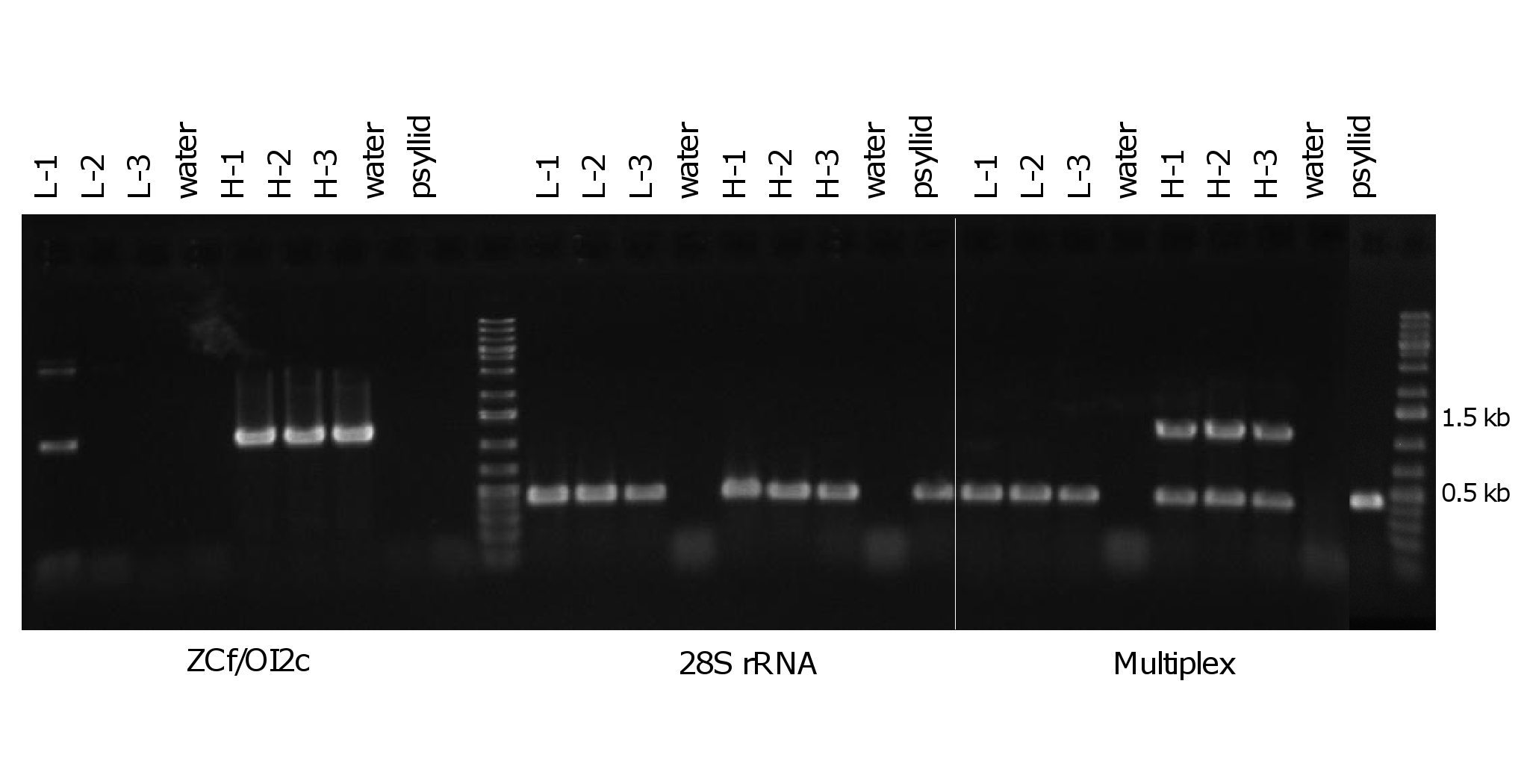

Supplement: Figure S2 — Analysis of CLs titer changes in psyllid populations. The prolificacy of the psyllid colony is a very good indication of the density of CLs in the psyllids. The high-CLs psyllid colony (H-1 through H-3) with low prolificacy was used to initiate a new high-CLs colony, on a fresh set of tomato plants. However in the process of adapting to the fresh tissue in a new cage, the colony started to rapidly proliferate and when evaluated by PCR the density of CLs was very low. Subsequently a simultaneous screening on both psyllid populations was performed. Three psyllids per colony were randomly picked and DNA was extracted, including a “water” DNA extraction within sets to account for any possible contamination during the extraction. Conventional PCR was conducted with primer pairs Zcf/OI2c and 28SrDNA, used in a single PCR or combined in a multiplex. Results show that the prolific colony has reduced CLs titer (L-1 to L-3) and the initial high-CLs colony (H-1 to H-3) retained elevated amounts of CLs. Moreover when multiplex PCR was performed, only the high-CLs colony yielded comparable results, amplifying both PCR products, but the low-CLs sample did not produce a PCR product for the Zcf/OI2c primer pair, implicating that the primer ratios and conditions for multiplex PCR when testing low titer colonies need to be adjusted. Size markers in Kb. (TIF) [file pone.0037345.s002.tif]

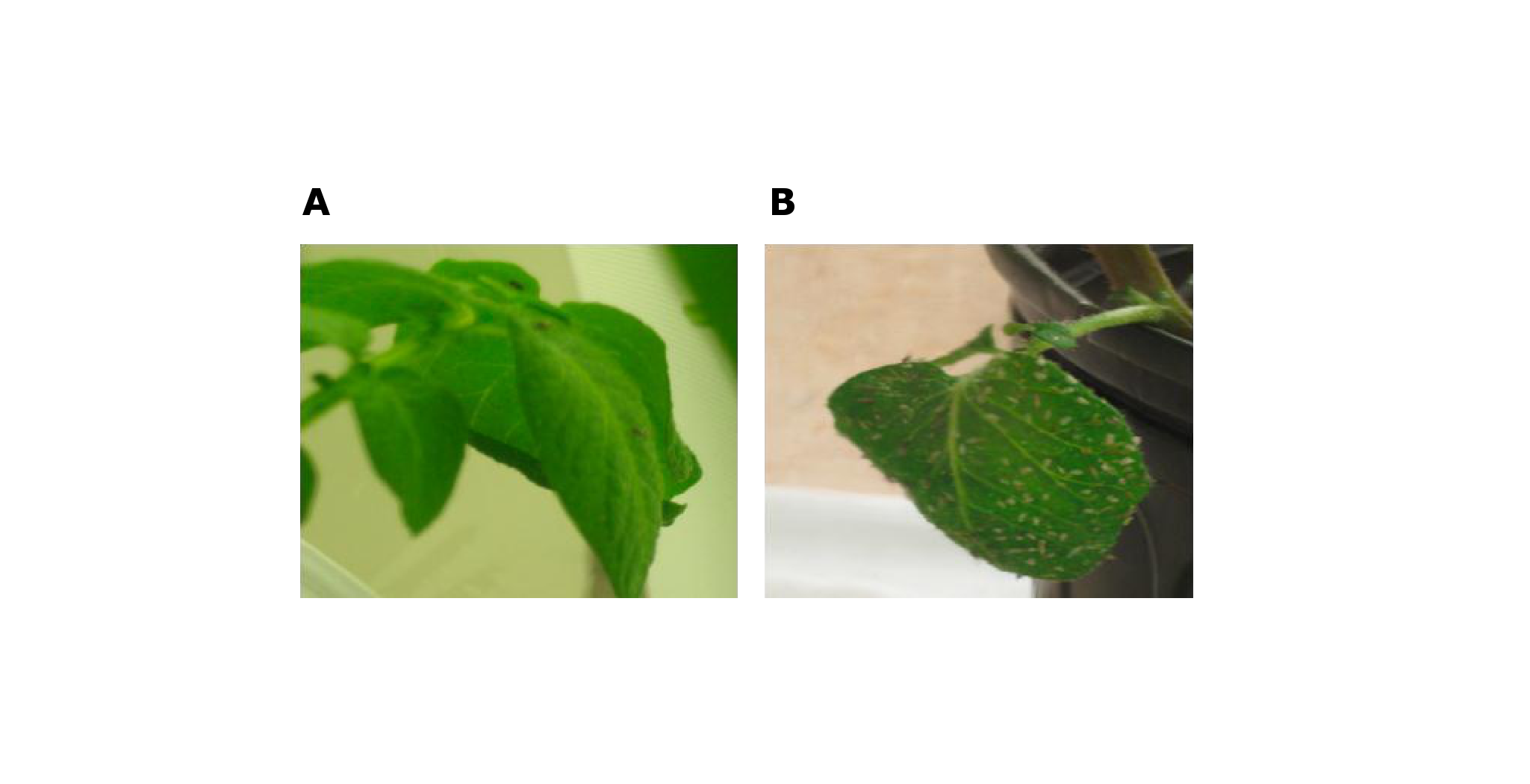

Supplement: Figure S3 — Prolificacy differences between psyllid colonies. New colonies were established on potato plants with 5 pairs of female and male psyllids, after a month substantial differences in the population numbers were observed. A. High-CLs C3 colony, B. Low-CLs C1 colony. (TIF) [file pone.0037345.s003.tif]

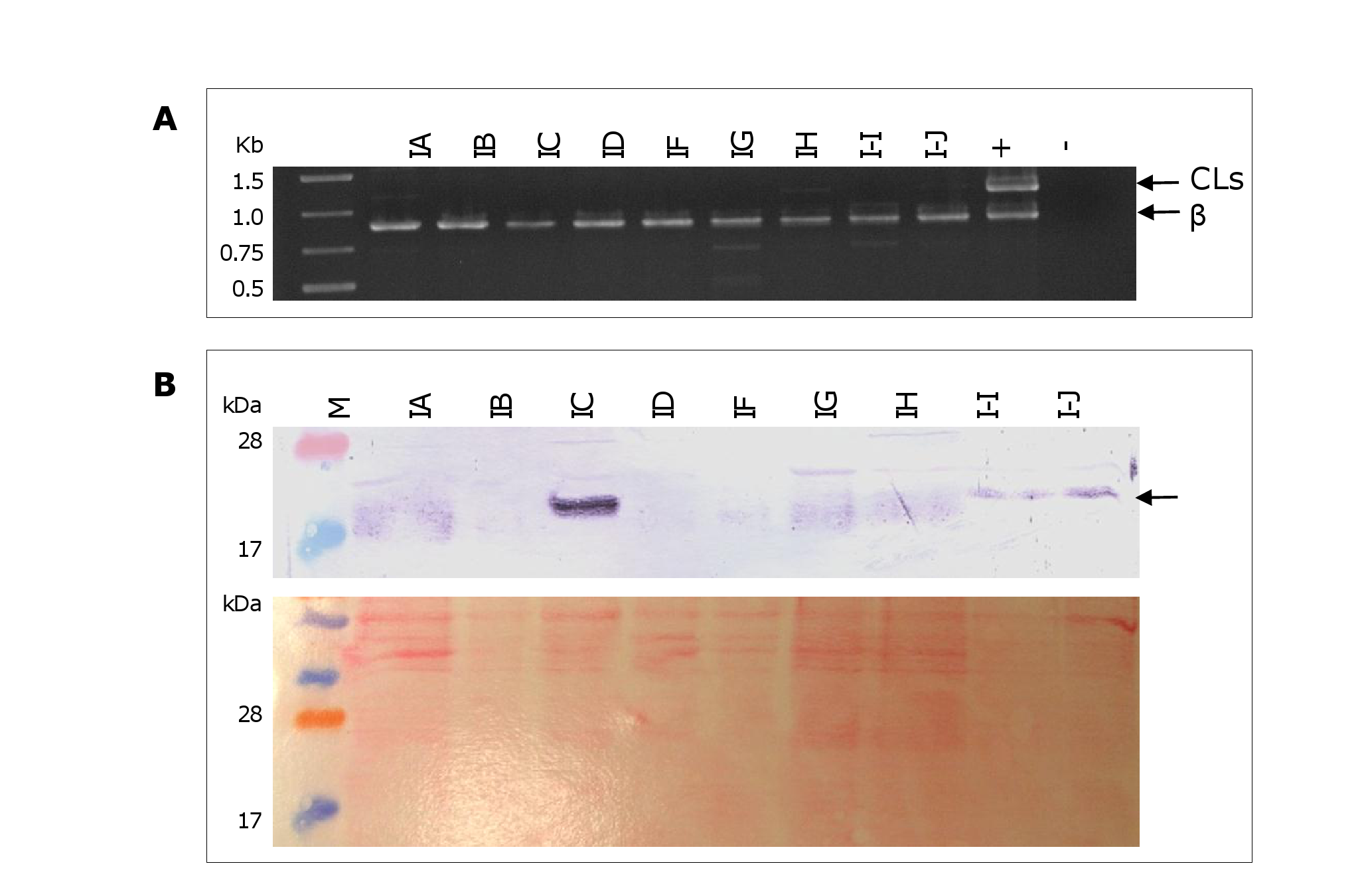

Supplement: Figure S4 — Molecular characterization of plants exposed to low-CLs psyllids. A. Multiplex PCR was conducted on DNA extracted from stems of plants exposed to low-CLs psyllids. Arrows indicate the position of the 1,171 bp CLs and the 881 bp β-tubulin (β) amplicons. β-tubulin is used as a marker for DNA quality control, B. Total protein was extracted from the same tissues and western blot analysis was performed for the detection of cyclophilin (arrow). Sample loading is shown by the red Ponceau S staining. Molecular markers are indicated. (TIF) [file pone.0037345.s004.tif]

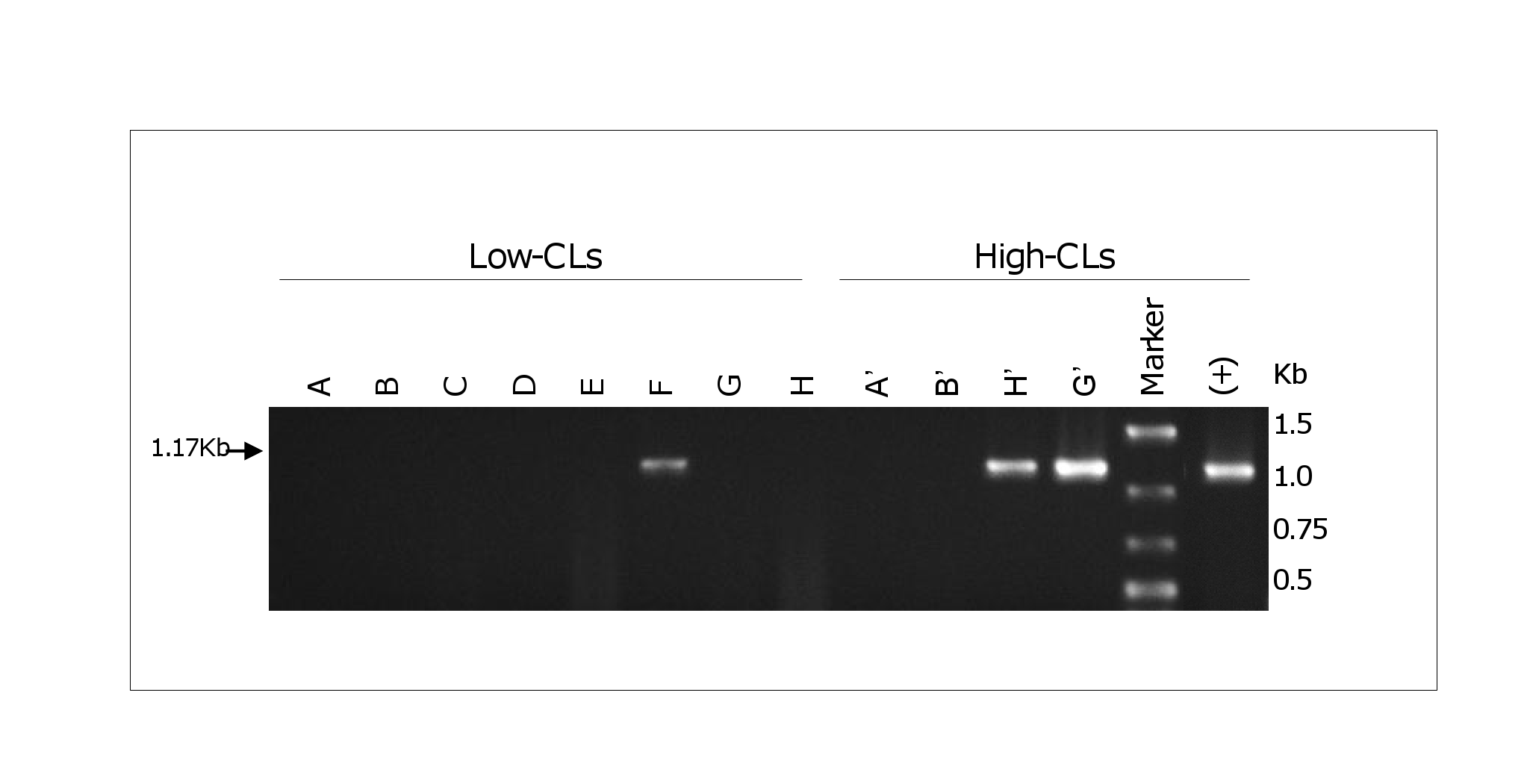

Supplement: Figure S5 — Conventional PCR screening of plant samples exposed to low and high-CLs psyllids. Conventional PCR tests were performed on DNA extracted from stems of plants exposed for 2.5 weeks to low-CLs (C1) and high-CLs (C2) psyllids. The arrow indicates the position of the 1.17 Kb ZCf/OI2c 16S rDNA amplicon. For the group of plants exposed to low-CLs, DNA samples A and B are from control plants that were caged without psyllids, and C through H represent plants exposed to psyllids, For plants exposed to high-CLs, A′ and B′ are DNA from control plants (caged without psyllids) and H′ and G′ are from plants exposed to high-CLs C2 psyllids. DNA ladder is indicated in kilo bases (Kb). Unnecessary lanes were removed (two lanes between marker and ZC plant DNA used as positive control). (TIF) [file pone.0037345.s005.tif]
