# Supplementary material for: Molecular and Physiological Properties Associated with Zebra Complex Disease in Potatoes and Its Relation with Candidatus Liberibacter Contents in Psyllid Vectors
Source: PLoS One. 2012 May 17;7(5):e37345. doi: 10.1371/journal.pone.0037345 (PMC3355140; doi:10.1371/journal.pone.0037345)
Supplement: Table S1 — RT-qPCR primers. (DOCX) [file pone.0037345.s006.docx]

**Supplemental Table 1.** RT-qPCR primers

| **Primer** | **Sequence** |
| --- | --- |
| 28S-Bc-F | TCGGTCGTTTCCGTTGGT |
| 28S-Bc-R | CAACATCACGCCCGAAGAC |
| 23-16S-F | ATACTGCCCAAGAGTCCATATCG |
| 23-16S-R | TGTGATGAGCCGACATCGA |
| ZCf | CGAGCGCTTATTTTTAATAGGAGC |
| HLBr | GCGTTATCCCGTAGAAAAAGGTAG |
| Vent8-F | AGCTTTATGCTGGTGCTCGTTAT |
| Vent8-R | CATCATCTTTAGCTGCCTTACCAA |
| Vent1-F | AGCAAAAGCTGGTGTTAGCTATGA |
| Vent1-R | CGAAGTAACGAGCTCCAGCAA |
